# Neuroprotective Effect of Transplanted Human Embryonic Stem Cell-Derived Neural Precursors in an Animal Model of Multiple Sclerosis

**DOI:** 10.1371/journal.pone.0003145

**Published:** 2008-09-05

**Authors:** Michal Aharonowiz, Ofira Einstein, Nina Fainstein, Hans Lassmann, Benjamin Reubinoff, Tamir Ben-Hur

**Affiliations:** 1 The Hadassah Human Embryonic Stem Cells Research Center, The Goldyne Savad Institute of Gene Therapy, Hadassah-Hebrew University Medical Center, Jerusalem, Israel; 2 Department of Neurology, The Agnes Ginges Center for Human Neurogenesis, Hadassah-Hebrew University Medical Center, Jerusalem, Israel; 3 Department of Gynecology, Hadassah-Hebrew University Medical Center, Jerusalem, Israel; 4 Center for Brain Research, Medical University of Vienna, Vienna, Austria; City of Hope Medical Center and Beckman Research Institute, United States of America

## Abstract

**Background:**

Multiple sclerosis (MS) is an immune mediated demyelinating disease of the central nervous system (CNS). A potential new therapeutic approach for MS is cell transplantation which may promote remyelination and suppress the inflammatory process.

**Methods:**

We transplanted human embryonic stem cells (hESC)-derived early multipotent neural precursors (NPs) into the brain ventricles of mice induced with experimental autoimmune encephalomyelitis (EAE), the animal model of MS. We studied the effect of the transplanted NPs on the functional and pathological manifestations of the disease.

**Results:**

Transplanted hESC-derived NPs significantly reduced the clinical signs of EAE. Histological examination showed migration of the transplanted NPs to the host white matter, however, differentiation to mature oligodendrocytes and remyelination were negligible. Time course analysis of the evolution and progression of CNS inflammation and tissue injury showed an attenuation of the inflammatory process in transplanted animals, which was correlated with the reduction of both axonal damage and demyelination. Co-culture experiments showed that hESC-derived NPs inhibited the activation and proliferation of lymph node–derived T cells in response to nonspecific polyclonal stimuli.

**Conclusions:**

The therapeutic effect of transplantation was not related to graft or host remyelination but was mediated by an immunosuppressive neuroprotective mechanism. The attenuation of EAE by hESC-derived NPs, demonstrated here, may serve as the first step towards further developments of hESC for cell therapy in MS.

## Introduction

Multiple sclerosis (MS), the most common cause of neurological disability in young adults, is a chronic, multifocal disease of the CNS. The pathological hallmarks of MS include immune cell infiltrations, oligodendrocyte death, demyelination and axonal damage [1]–[3]. Failure of the CNS to remyelinate MS lesions [4] and axonal damage [5], [6] lead to the irreversible functional decline of MS patients [7], [8].

While cell transplantation therapy of MS was initially suggested for oligodendroglial cell replacement and myelin regeneration [9], recent studies have focused on the anti-inflammatory effects of neural precursor cells (NPs). Transplantation of mouse brain-derived NPs into the CNS of rodents with experimental autoimmune encephalomyelitis (EAE), the animal model of MS, attenuated the brain inflammatory process and severity of clinical disease [10]–[12]. Since demyelination and acute axonal injury in MS are considered to result mainly from the acute inflammatory process, it raises the notion that the neuroprotective effect of transplanted NPs in EAE is mediated, at least in part, through the attenuation of the inflammatory process and prevention of its secondary neurodegenerative effects.

Human embryonic stem cells (hESC) may potentially serve as an unlimited source of neural cells for transplantation in neurological disorders, such as MS. Here we transplanted hESC-derived NPs into the cerebral ventricles of EAE mice. We show here for the first time that transplantation of hESC-derived NPs attenuates the clinical signs of EAE and reduces CNS inflammation and tissue injury. Moreover, we show that the therapeutic effect of the transplanted NPs was not mediated by graft- or host-derived remyelination, but by the suppression of the acute phase of the inflammatory process in the transplanted- EAE animals protecting them from chronic neurological residua. We therefore conclude that transplanted hESC-derived NPs exert a neuroprotective effect on the CNS of EAE mice.

## Results

### hESC-derived NPs express markers of neural precursor cells and differentiate *in-vitro* mainly into neurons and astrocytes

**Figure 1:**
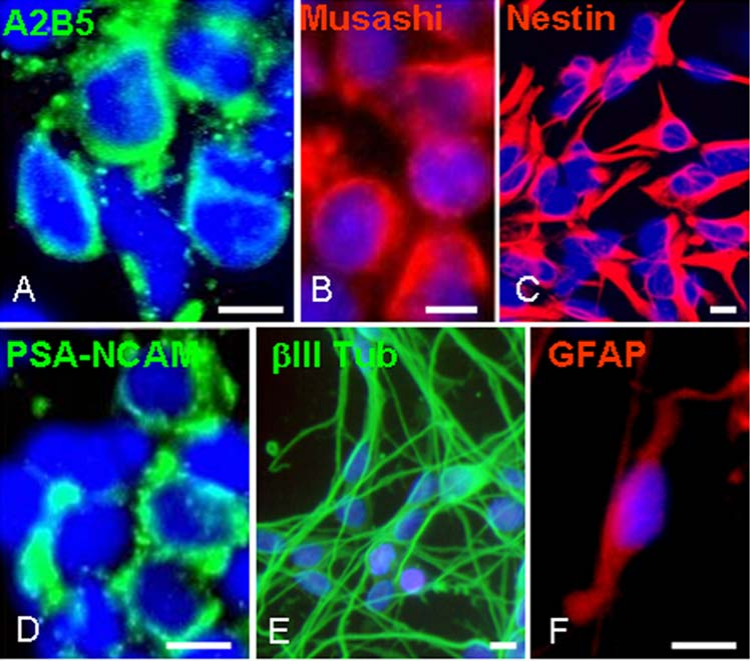
Characterization of the hESC-derived spheres prior to transplantation. Spheres enriched for NPs were developed from hESCs that were cultured for 3 weeks as free floating clusters in serum-free medium supplemented with BFGF, EGF and noggin. The spheres were further expanded for 4–6 weeks in the same medium supplemented with bFGF and EGF before transplantation. For *in-vitro* characterization spheres were triturated and plated on fibronectin-coated coverslips. Immunofluorescent stainings, 2 hours after plating, demonstrated that the majority of the cells within the spheres were immunoreactive with anti-A2B5 (A), -Musashi (B), -Nestin (C) and -PSA-NCAM (D). To induce differentiation, the plated cells were further cultured 7 days in the absence of mitogens. Immunofluorescent stainings showed that the NPs differentiated mainly into βIII tubulin-expressing neurons (E) and GFAP expressing astrocytes (F). Cells expressing oligodendroglial markers were not observed. Nuclei in A–F are counterstained with DAPI (blue). Scale bars: 15 µm.

Highly enriched cultures of NPs were derived from hESCs and propagated as spheres according to our previously published protocol [13]. Briefly, clusters of hESCs were cultured as floating aggregates in defined medium, supplemented with the bone morphogenetic protein antagonist, noggin, basic fibroblast growth factor (bFGF) and epidermal growth factor (EGF). Under these culture conditions the cell-clusters developed into spheres that were mainly comprised of NPs. After 7–9 weeks of propagation, prior to transplantation into the brains of EAE mice, we characterized the phenotype and *in vitro* differentiation potential of the cells within the spheres. The spheres were highly enriched with uncommitted NPs, as indicated by the expression of nestin, polysialylated neural cell adhesion molecule (PSA-NCAM), Musashi and A2B5 by over 90% of the cells (Fig. 1). When the human neural sphere cells were plated on fibronectin, in the absence of growth factors, and allowed to differentiate for seven days, they gave rise mainly to neurons and astrocytes, as indicated by the expression of the neuronal marker βIII tubulin and the astrocyte marker glial fibrillary acidic protein (GFAP) by 67±9% and 12±4% of the differentiating cells respectively (Fig. 1). Cells expressing the oligodendroglial markers, O4 and galactocerebroside (GalC) were not observed.

### The clinical course of EAE is significantly milder in NP-transplanted as compared to control mice

**Figure 2:**
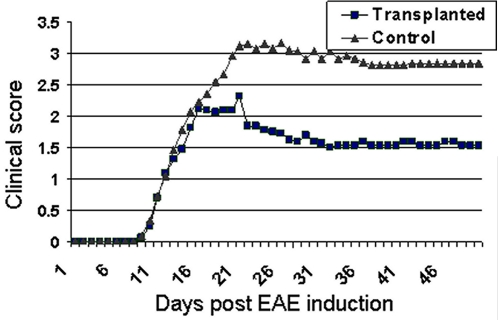
The clinical course of EAE is attenuated after transplantation of hESC-derived NPs. hESC-derived NPs were transplanted into the lateral brain ventricles of EAE mice and the severity of clinical signs was scored daily. A significant attenuation of the clinical score was observed in NPs-transplanted animals (▪) in comparison to vehicle-transplanted control animals (▴). The reduced severity of clinical scores in NP-transplanted animals was evident as early as the acute phase of the disease (day 19 post-EAE induction). See material and methods section for details regarding the clinical scoring system.

EAE was induced in 6–7 week old female C57B/6 mice by immunization with purified myelin oligodendrocyte glycoprotein (MOG) peptide as previously described [14]. Seven days after EAE induction, hESC-derived neural spheres were engrafted after partial disaggregation into the lateral brain ventricles of the MOG EAE mice (n = 15). In some experiments the spheres were derived from green fluorescent protein (GFP) expressing hESCs to facilitate the identification of transplanted cells *in vivo*. The spheres' culture medium was transplanted into the ventricles of control EAE mice (n = 21). Both animal groups received cyclosporine daily from the day of transplantation. In preliminary experiments we demonstrated that daily cyclosporine treatment, with a dose that was shown to be effective in preventing graft rejection [15] had no effect on the clinical and pathological course of MOG EAE (data not shown). The NPs and vehicle transplanted mice were scored daily for clinical signs of EAE during a 43 day period (Fig. 2).

Statistical analysis of the clinical scores revealed that the clinical signs of EAE were significantly attenuated in NP-transplanted as compared to control EAE mice. Both the maximal clinical scores and the cumulative scores were significantly reduced in the NP-transplanted animals (Table 1).

**Table 1:**
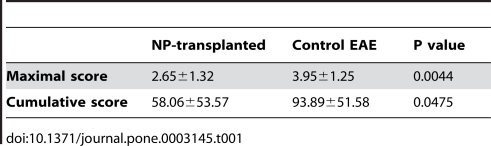
Transplantation of hESC-derived NPs significantly attenuates the clinical severity of EAE.

### Transplanted NPs differentiate in EAE brains mainly into early immature neural cells and do not contribute to remyelination

**Figure 3:**
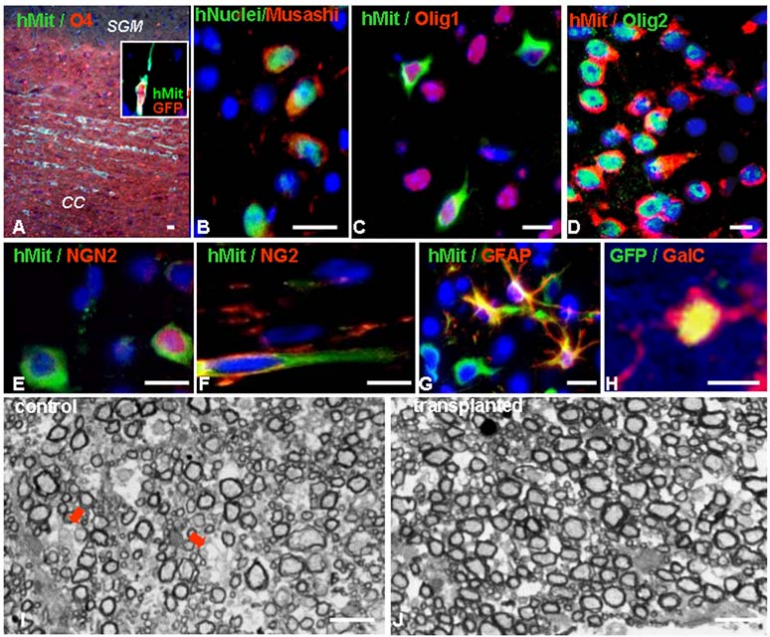
Transplanted NPs migrate into white matter tracts of EAE brains, differentiate into neural progenitors and do not promote remyelination. (A–H) Immunofluorescence stainings of brain sections demonstrating the migration and differentiation of transplanted NPs, which were identified by the expression of human mitochondria (A, C–G), human nuclear antigens (B) and GFP (inset in A, H). (A): The NPs migrated extensively into white matter areas of the CNS such as the corpus callosum (*CC*) and were not observed in grey areas such as subcortical grey matter (*SGM*). Co-staining against the oligodendroglial marker, O4 (red) was used to identify the white matter. (B–H): Most of the transplanted cells either remained as uncommitted NPs expressing Musashi (B) or differentiated into early neuronal/oligodendroglial progenitors expressing Olig1 (C) or Olig2 (D). Further differentiation into more committed neuronal progenitors, oligodendrocyte progenitors or astrocytes expressing NGN2 (E), NG2 (F) and GFAP (G), respectively, was infrequent (∼1% of the transplanted cells per each of the cell types). Terminal differentiation of the transplanted NPs into GalC-expressing mature oligodendrocytes (H) was rare (<0.01% of the transplanted cells). Nuclei in A–H are counterstained with DAPI (blue). I–J: Toluidine blue stained transverse semi-thin sections of resin embedded spinal cords of NP-transplanted (J) and control (I) animals. In NP-transplanted animals, there were less demyelinated (arrows in I) and more normally myelinated axons than in controls. In both groups there were very rare remyelinated axons (G ratio>0.8). Scale bars: 15 µm (A–H), 5 µm (I–J).

Following the 43 day period of clinical follow up, the study and control animals were sacrificed for histopathological analysis. The transplanted NPs were identified in brain sections by immunofluorescent stainings for human-specific mitochondria, human specific nuclear antigens or GFP (Fig. 3). The engrafted human cells survived and migrated extensively from the lateral ventricles into white matter areas such as the corpus callosum and the periventricular white matter, where an inflammatory process was observed. They were not detected in grey matter areas such as the subcortical grey matter (Fig. 3A).

Serial hematoxylin and eosin (H&E)-stained sections covering the entire brain did not reveal teratoma tumors in the transplanted mice

We further characterized the differentiation of the transplanted cells in the CNS. The vast majority of the transplanted human NPs either remained as uncommitted precursors (33±6%), identified by the expression of the RNA binding protein, Musashi (Fig. 3B) or committed into common bipotential neuronal/oligodendroglial progenitors, expressing the basic helix-loop-helix (bHLH) transcription factors olig1 (28±4%) and olig2 (31±12%) (Fig. 3C, D). Some transplanted cells further differentiated into oligodendroglial progenitors, expressing NG2 (Fig. 3F), neuronal progenitors expressing Ngn2 (Fig. 3E) and astrocytes expressing GFAP (Fig. 3G). The incidence of differentiation into these three cell types was ∼1% per cell type. However, the differentiation of the transplanted NPs into mature oligodendrocytes expressing markers such as GalC (Fig. 3H) was extremely rare (<0.01%).

The extremely limited differentiation of the transplanted NPs into mature oligodendrocytes *in-vivo* suggested that their therapeutic effect in EAE was not mediated by graft-derived remyelination. To determine whether transplantation had an effect on endogenous remyelination we calculated the G ratios (axon diameter/axon+myelin sheath diameter) of axons from toluidine blue stained spinal cord semi-thin sections from animals that were sacrificed 50 days post EAE induction. Axons with a G ratio>0.8 were considered remyelinated [16], [17]. Analysis of the G ratios values of axons from the NP-transplanted and control groups (n = 5 per group) revealed low percentages (<1%) of remyelinated axons in both groups (Fig. 3I, J). These findings suggested that the clinical improvement that was observed in transplanted animals was not related to enhanced remyelination neither by donor nor host oligodendroglial cells.

### Transplanted hESC-derived NPs attenuate the inflammatory process and the evolution of host tissue damage

Given the lack of evidence for myelin regeneration after transplantation, we hypothesized that the beneficial effect of the transplanted NPs was mediated through a neuroprotective mechanism. We and others have recently shown an immunosuppressive effect of transplanted, rodent brain-derived NPs that was accompanied by attenuation of tissue damage in the CNS of EAE animals [11], [12], [18]. To determine whether the transplanted hESC-derived NPs protected the CNS from the detrimental effect of EAE, by suppressing inflammation and its consequent damage to the CNS we performed a time course experiment in which we followed the evolution of the inflammatory process and the tissue damage and compared it between NP-transplanted and control EAE mice. Following induction of MOG EAE and transplantation, as described above, mice from NP-transplanted and control groups were sacrificed at the following 4 time points, which represented 4 critical stages in the course of EAE [19]: day 10 in which immune cells begin to infiltrate the CNS although the disease does not yet manifest clinically; day 13 in which there are early clinical signs and inflammation is more robust; day 20 which represents the peak of the acute phase of MOG EAE; and day 50 which represents the chronic phase. In each time point (n = 4–5/group per time point) we performed histochemical and pathological analysis of spinal cord sections to quantify the severity of inflammation, demyelination and axonal damage.

To measure the extent of inflammation, we examined the numbers of immune cell infiltrations, CD3+ T cells and Mac3+ macrophages/activated microglia per mm^2^ of the sections. We measured the extent of demyelination and axonal injury by analyzing the percentage of area of the spinal cord sections that was not stained by luxol fast blue (LFB) and Bielschowsky's reagents, respectively.

**Figure 4:**
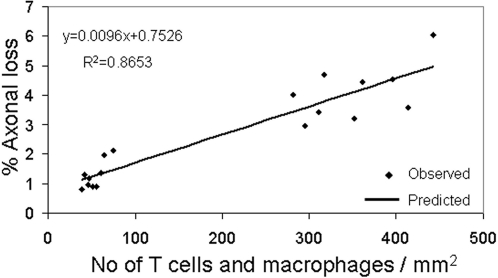
The extent of the inflammatory process strongly correlates with the severity of tissue damage in the acute phase of EAE. NP- transplanted and control mice were sacrificed at 10, 13, 20 and 50 days post EAE induction for pathological analysis of inflammation and tissue damage. Linear-regression analysis of the numbers of inflammatory cells and the extent of axonal loss in both NP-transplanted and control EAE mice at 13 and 20 days post EAE induction showed a strong correlation between the numbers of T cells and macrophages per mm^2^ and the percentage of axonal loss.

We initially assessed the association between inflammation and tissue damage at the acute phase (days 13 and 20) of MOG EAE by plotting the % of axonal loss against the numbers of CD3+ cells and Mac3+ cells/mm^2^ in NP-transplanted and control mice (Fig. 4). Regression analysis demonstrated a strong correlation between the amount of the inflammatory cells and the severity of tissue damage (R^2^ = 0.86), in the acute phase of EAE (Fig. 4).

**Figure 5:**
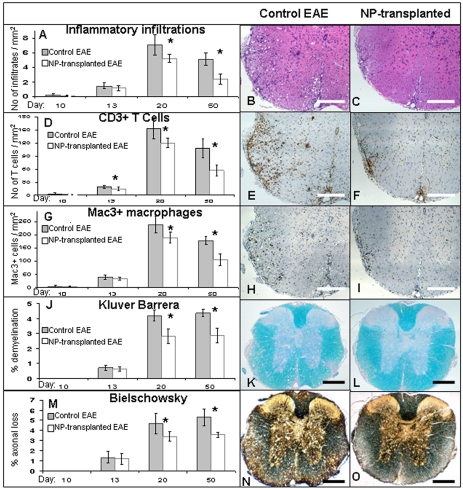
Attenuation of the progression of inflammation and tissue damage in the CNS of NPs-transplanted mice. Pathological examination of spinal cord sections from NP-transplanted and control mice were performed at 10, 13, 20 and 50 days post EAE induction to evaluate CNS inflammation, demyelination and axonal damage. In NP-transplanted mice an attenuation of the number of immune-cell infiltrates (A), T cells (D) and macrophages/activated microglia (G) per mm^2^ was evident from day 13 post EAE induction and became significant at days 20 and 50. Demyelination and axonal damage, which were analyzed by loss of Kluver Barrera (J), and Bielschowsky staining (M), respectively, were both significantly reduced at day 20 and 50 post EAE induction. The differences between the study and control groups in the severity of all parameters gradually increased with time. Representative day 20 images of H&E staining (B, C), immunostaining for CD3 (E, F) and Mac3 (H, I), Kluver Barrera staining (K, L) and Bielschowsky silver staining (N, O). * P<0.05. Scale bars: 100 µm.

Analysis of the evolution of inflammation and tissue damage showed initial infiltration of immune cells into the CNS in both groups as early as 10 days post EAE induction. At this stage there was no histological evidence of demyelination or axonal injury (Table 2). At day 13, further infiltration of immune cells was observed in both groups, however, the numbers of CD3+ cells/mm^2^ was significantly reduced in the NP-transplanted in comparison to the control animals. At this stage, demyelination and axonal injury were still minimal and similar in both groups (Fig. 5J, M, Table 2). At day 20, which represents the peak of the acute phase of MOG EAE, both the numbers of CD3+ cells/mm^2^ and Mac3+ cells/mm^2^ as well as the number of immune cell infiltrations/mm^2^ were significantly decreased in the NPs-transplanted animals (Fig. 5A, D, G, Table 2). The differences between the groups in the numbers of immune cells in the CNS further increased and were even more prominent at the latest time point that we examined (day 50; Table 2). In parallel, axonal damage and demyelinantion became significantly reduced in the NP-transplanted group as compared to the controls at day 20 post induction and the difference between the groups in the amount of axonal damage even increased at the last time point we examined (Fig. 5J, M, Table 2).

**Table 2:** Histopathological analyses of inflammatory parameters, demyelination and axonal damage in the spinal cord of C57BL/6 mice at 10, 13, 20 and 50 days after MOG35–55 EAE induction.

	Control EAE	NP-transplanted	P value
**Infiltrations/mm^2^**			
**day 10**	0.18±0.2	0.1±0.13	0.5
**day 13**	1.43±0.5	1.15±0.4	0.32
**day 20**	7.1±1.4	5.22±0.6	0.044
**day 50**	5.1±0.9	2.41±0.7	0.006
**T-cells/mm^2^**			
**day 10**	2.5±2.9	1.66±2.3	0.65
**day 13**	19.16±4.2	14.66±4.3	0.047
**day 20**	152.9±22.7	120.2±11.1	0.016
**day 50**	108.05±20.9	57.29±13	0.029
**Macrophages/mm^2^**			
**day 10**	4.79±6	3.5±4.8	0.73
**day 13**	40.2±8.5	34.16±5.8	0.24
**day 20**	240±32.9	190±19.8	0.027
**day 50**	179.44±15.9	106.04±22.5	0.005
**% Axonal injury**			
**day 10**	0	0	-
**day 13**	1.3±0.57	1.23±0.53	0.758
**day 20**	4.65±1	3.41±0.4	0.038
**day 50**	5.22±0.83	3.58±0.25	0.024
**% Demyelination**			
**day 10**	0	0	-
**day 13**	0.68±0.15	0.6±0.14	0.409
**day 20**	4.2±0.36	2.82±0.5	0.002
**day 50**	4.36±0.24	2.85±01.4	0.002

These time course experiments showed the initiation of the inflammatory process before the demonstration of CNS tissue injury. They also suggested that the attenuation of the acute inflammatory process of EAE by the transplanted NPs contributed to the decreased tissue damage in the CNS. Quantification of apoptotic CD3+ T cells in the histopathological sections showed 3.2±2.4% pyknotic T cell nuclei in control EAE CNS and 2.7±2.5% in transplanted CNS. Thus, the effect of transplantation was not mediated by induction of T-cell apoptosis in the CNS of EAE mice. The results of the time course experiments, together with the negligible occurrence of remyelination that was demonstrated above, suggest that neuroprotective rather than regenerative mechanisms underlie the therapeutic effect of transplantation.

### hESC-derived NPs inhibit activation and proliferation of lymph-node cells, in response to concanavalin A

**Figure 6:**
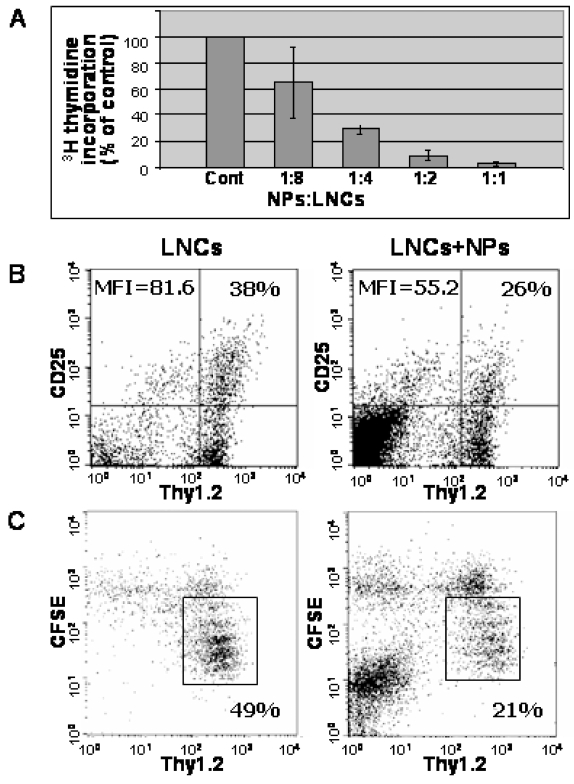
Direct suppressive effects of hESC-derived NPs on lymph node cells (LNCs) and T cells derived from naïve C57BL mice. LNCs derived from naïve mice were co-cultured with NPs and activated with ConA. The NPs suppressed ^3^H-thymidine incorporation into the activated LNCs in a dose-dependent manner (A). FACS analysis of interleukin-2 receptor α (IL-2Rα; CD25) expression after 24 hours of ConA-stimulation showed that human NPs inhibited the activation of Thy1.2+ T cells as determined by the fraction of labeled cells and by mean fluorescent intensity (MFI) (B). FACS analysis of CFSE labeled LNCs after 72 hours of ConA stimulation showed that co-culturing with the human NPs inhibited the proliferation of Thy1.2+ T cells. The fraction of T cells that have proliferated and therefore diluted the CFSE fluorescence (within the rectangle) was reduced in the presence of the human NPs (C).

We have shown previously that NPs derived from brains of newborn mice exhibited a bystander inhibitory effect on T-cell activation and proliferation *in vitro* [18]. Here, we analyzed whether the hESC-derived NPs have similar immunosuppressive properties by co-culturing them with lymph-node cells (LNCs). We first used the ^3^H-thymidine incorporation assay to test whether hESC-derived NPs exert a direct suppressor effect on the *in-vitro* proliferation of LNCs obtained from naïve C57BL mice. The human NPs inhibited LNC proliferation in response to concanavalin A (ConA), in a dose dependent manner (Fig. 6A). A maximal effect of 91% inhibition in ^3^H-thymidine incorporation was obtained when NPs/LNC ratio of 1∶2 was used. We therefore used this ratio for all the following experiments *in-vitro*.

We next investigated the effect of hESC-derived NPs on T-cell activation and proliferation. We measured the induction of IL-2Rα, a marker for T-cell activation, in Thy1.2+ T cells. In LNCs co-cultured with the human NPs the fraction of IL-2Rα+ T cells was reduced by 32%, and a similar decrease was observed in the mean fluorescence intensity of IL-2Rα (Fig. 6B). In addition, naive LNCs were labeled with CFSE and stimulated with ConA in the presence or absence of NPs. FACS analysis showed that the human NPs reduced the fraction of cycling T cells from 49% to 21% (Fig. 6C).

## Discussion

We show here that transplantation of hESC-derived NPs into the brain ventricles reduces the severity of clinical signs and tissue damage in the CNS of EAE mice. The beneficial effect is mediated by a neuroprotective rather than a regenerative mechanism. This is the first report of the therapeutic potential of NPs derived from human ESC in an animal model of MS.

The amelioration of EAE without differentiation of the transplanted cells into myelinating oligodendrocytes suggests that the beneficial effect of transplantation was not via cell replacement. The hESC-derived NPs used in our study, as opposed to neural precursors derived from postnatal brains, rarely differentiate into oligodendroglial cells, unless they are specifically directed to this fate *in-vitro* [20]. Thus, the negligible differentiation of the NPs into mature oligodendrocytes both *in-vitro* and *in-vivo* indicates that remyelination by the transplanted cells was very unlikely. Another mechanism by which transplanted cells might exert a therapeutic effect in EAE is by enhancing host remyelination. It was previously shown that NPs express a wide range of neurotrophic, and mitogenic factors, such as bFGF and platelet-derived growth factor (PDGF), which stimulate recruitment and proliferation of endogenous oligodendrocyte progenitors. Furthermore, NPs also express several factors that increase oligodendrocyte survival [21], [22]. However, our analysis showed that remyelination was rare, and could be demonstrated in less than 1% of the axons from both transplanted and control EAE groups. The very limited remyelinatin in MOG 35–55 EAE C57BL/6 mice is related to the extensive axonal injury in this specific model. Therefore, regenerative properties of hESC-derived NPs, whether directly or by inducing endogenous remyelination will need to be examined in other experimental models of MS. In summary, our findings indicate that the beneficial effect of transplantation was mediated by a non regenerative, neuroprotective mechanism.

Recent studies showed that transplanted rodent brain-derived NPs suppress CNS inflammation in EAE [10]–[12], and suggested that they exert immunosuppression either via a non-specific bystander inhibitory effect on T-cell activation [18] or through a specific immunomodulatory mechanism [12]. To demonstrate that suppression of inflammation and its consequent CNS tissue damage contributed to the neuroprotective effect that was observed in the present experiments, we studied here for the first time the progression of the inflammatory process and the tissue damage in a time course experiment. An initial step of the acute inflammatory process in EAE is T-cell infiltration which triggers macrophage invasion and microglia activation. We showed a significant reduction in the number of infiltrating T-cells in the spinal cord of NP-transplanted animals at an early stage of the acute phase of EAE, when tissue damage was minor and similar in both study and control groups. This was followed by a significant attenuation of the acute and chronic inflammatory processes in the NP-transplanted mice, which correlated with a significant reduction in the acute tissue damage and subsequent chronic tissue loss. Therefore, this longitudinal study provides a potential link between the immunosuppressive effect of the transplanted NPs and the protection from tissue injury in MOG35–55 EAE. We further analyzed the immunosuppressive effect of the human NPs in co-culture experiments. These indicated a generalized bystander inhibitory effect of the human NPs on T cell activation and proliferation, similar to the effect of rodent brain derived neural precursors [18]. Further studies are required to determine the molecular mechanisms that underlie the immunosuppressive effects of hESC-derived NPs and whether they are shared with other cell types.

Cell therapy in MS is rapidly approaching a clinical phase. Candidates include bone marrow stromal cells [23], adult NPs [9], [12] and hESC-derived NPs. We have recently shown that hESC-derived NPs migrate efficiently and integrate well in white matter tracts of EAE mice, similar to rodent NPs [24]. This property and the ability of NPs to appropriately respond to the micro-environment at the site of transplantation [25] may be beneficial for their long term survival and function, as compared to non-neural cells. In addition, protocols to direct the differentiation of hESC into oligodendroglial cells were recently established, enabling them to be utilized as a source of remyelinating cells in myelin basic protein (MBP)-deficient shiverer mice [26], [27] and in a model of focal spinal cord lesion [28]. Therefore, combining the anti-inflammatory effect of hESC-derived NPs with the remyelinating potential of oligodendroglial cells derived from hESC may provide an advantage to hESC-based cell therapy over existing conventional immunosuppressive/immunomodulatory treatments, as well as over non-neural sources of cells for transplantation in MS.

In conclusion, this study is the first to demonstrate a beneficial anti-inflammatory and neuroprotective effect of hESC- derived NPs leading to clinical and pathological amelioration in a relevant model of MS. These data may serve as the platform for further developments that may eventually allow the use of hESC for transplantation in MS.

## Materials and Methods

***hESC culture:*** hESC (HES-1 cell line) with a stable normal (46XX) karyotype were cultured on human foreskin feeders in serum free medium as described [29] and were passaged weekly by treatment with collagenase IV (1 mg/ml for 20 min at 37°C).

***Generation of highly enriched populations of NPs for transplantation:*** wild type and cloned genetically-modified hESCs that were infected by a lentiviral vector expressing eGFP under the human EF1α promoter [30] were used for derivation of NPs for transplantation into EAE mice. Colonies of undifferentiated hESCs were removed from the feeders by treatment with collagenase IV (1 mg/ml for 20 min at 37°C), transferred to 24-well culture dishes (Costar; Corning, Inc., Corning, NY, USA), and cultured in suspension in a chemically-defined neural precursor medium (NPM) consisting of DMEM/F12 (1∶1), B27 supplement (1∶50), 2 mM glutamine, 50 units/ml penicillin, 50 μg/ml streptomycin (Gibco), 20 ng/ml rh-bFGF, and 20 ng/ml rh-EGF (both from R&D Systems Inc., Minneapolis, MN). Recombinant mouse noggin (700 ng/ml; R&D) was added to the NPM to promote neural differentiation as described [13]. After three weeks under these culture conditions the neural spheres that developed were further expanded in NPM bFGF and EGF in the absence of noggin, for 5 more weeks before transplantation.

***Animals:*** For MOG EAE induction and transplantation experiments, 6–7 week old C57BL female mice were supplied by Harlan laboratories and were maintained in a specific pathogen free (SPF) unit.

***MOG EAE induction:*** EAE was induced in 6–7 week old female C57BL mice by immunization with an emulsion containing 300 μg of purified MOG peptide (MEVGWYRSPFSRVVHLYRNGK, corresponding to residues 35–55) in PBS and an equal volume of complete Freund's adjuvant containing 5 mg H37RA (Difco). 0.2 ml of the inoculum was injected subcutaneously at day of induction (day 0) and at day 7. In addition, 300 ng of Bordetella pertusis toxin (Sigma) in 0.2 ml PBS was injected intraperitoneally at day of induction and at day 2.

***Clinical evaluations of EAE:*** After EAE induction, mice were scored daily for EAE clinical signs, according to the following score: ***0***, asymptomatic; ***1***, partial loss of tail tonicity; ***2***, atonic tail; ***3***, hind-leg weakness and/or difficulty in rolling over; ***4***, hind-leg paralysis; ***5***, four-leg paralysis; ***6***, death due to EAE.

At the end of the follow-up period, the maximal score and the cumulative score of each animal were calculated. Maximal clinical score was calculated as the mean of the maximal clinical scores during the experimental period. Cumulative clinical score was calculated as the mean of the sum of the daily clinical scores during the experimental period.

***Transplantation of NPs:*** Seven days post EAE induction (day 7) the mice were anesthetized with intraperitoneal injection of pentobarbital (0.6 mg/10 gr) and were fixed in a stereotactic device. Quantities of 5×10^5^ cells or NPM in a volume of 7.5 μl were injected into each lateral ventricle.

***Tissue fixation and histological preparation:*** For analysis of the *in-vivo* localization and differentiation of the transplanted cells and for analysis of remyelination, EAE animals were sacrificed at the end of the follow-up period (50 days post-EAE induction). For histopathological analysis of the progression of inflammation and tissue damage in the time course experiment animals were sacrificed at 10, 13, 20 and 50 days post EAE induction (n = 4–5 per group at each time point). Animals were anesthetized with a lethal dose of pentobarbital and brains and spinal cords were perfused via the ascending aorta with ice-cold PBS followed by cold 4% paraformaldehyde (PFA) in PBS. The tissues were dissected and post-fixed by immersion in the same fixative for 24 h at 4°C. Brains were deep frozen in liquid nitrogen and cut to serial 6–8 μm axial and longitudinal sections and spinal cords were embedded in paraffin for pathological analysis.

***Pathological analysis:*** Analysis of inflammation, demyelination and axonal damage was performed on 5 μm paraffin-embedded serial transverse sections in three different rostrocaudal levels of the spinal cord. For histochemical analysis, sections were stained with H&E, LFB/periodic-acid Schiff staining, and Bielschowsky silver impregnation to assess inflammation, demyelination, and axonal pathology, respectively. In adjacent serial sections, immunohistochemistry was performed with antibodies against macrophages/activated microglia (rat anti-mouse Mac3, 01781D, clone M3/84; 1∶200; Pharmingen, San Diego, CA) and T cells (rat anti-human CD3, MCA 1477; 1∶400, Serotec, Bicester, United Kingdom). Primary antibodies were detected by the avidin-biotin technique using biotin conjugated secondary antibodies. The total average number of positive cells per square millimeter, in spinal cord cross sections, was counted using a grid overlay.

Apoptosis of T cells in the CNS was determined morphologically by the appearance of condensed and fragmented nuclei in CD3+ cells. The percentage of apoptotic cells was determined in transplanted and control animals (n = 3 in each group) by morphological analysis of 250 CD3+ cells in random CNS sections.

Demyelination and axonal damage were assessed in spinal cord sections by calculating the area of LFB and Bielschowsky silver staining loss, representing areas of myelin destruction and axonal loss, respectively. The percentage of demyelinated and axonal damage areas was determined by counting intersections of the grid over the demyelinated lesions and the areas of axonal loss.

For the evaluation of remyelination, animals were perfused with 4% gluteraldehyde. The fixed spinal cords were cut into 1 mm transverse blocks from the cervical, thoracic and lumbar areas. The blocks were osmicated, dehydrated through an ascending series of ethanols and embedded in TAAB resin. 1 μm sections were cut from each block, stained with toluidine blue (Sigma) and examined by light microscopy.

To determine whether transplantation had an effect on remyelination, the diameter of axons from toluidine blue stained spinal cord semi-thin sections was measured, and their G ratios were calculated (G = axon diameter/(axon+myelin sheath diameter)). The G ratio of intact axons is 0.5–0.8. Since the myelin sheath is thinner in remyelinated axons, an axon with a G ratio>0.8 was considered remyelinated.

***Immunofluorescent staining of NPs in vitro and in vivo:*** The following primary antibodies were used: Rabbit IgG anti-GFP (1∶100, Chemicon), mouse IgG anti-human specific mitochondria (1∶200, Chemicon), mouse IgM anti-A2B5 (1∶1, ATCC), mouse IgM anti-PSA-NCAM (1∶200, Chemicon), rabbit IgG anti-nestin (1∶50, Chemicon), rabbit anti-musashi (1∶100, Chemicon), mouse IgG anti-human nuclei (1∶50, Chemicon), rabbit IgG anti-NG2 (1∶50, Chemicon), mouse IgM anti-PDGFRα (1∶20, R&D), rabbit IgG anti-NGN2 (1∶300, Chemicon), rabbit anti-GalC (1∶20, Chemicon), mouse IgM anti-O4 (1∶20, Chemicon), rabbit IgG anti-MAP2 (1∶200, Chemicon), mouse IgG anti-β tubulin III (1∶2000, Sigma), rabbit anti-GFAP (1∶100, Dako), rabbit IgG anti-olig1 (1∶20, Chemicon) and goat anti-olig2 (1∶30, R&D). Texas red or Alexa 488-conjugated goat anti-mouse IgM (1∶100, Jackson, West Grove, PN), goat anti-rabbit IgG (1∶100, Molecular Probes), goat anti-mouse IgG (1∶100, Molecular Probes) or donkey anti-goat IgG (1∶200, Jackson, West Grove, PN) were used as secondary antibodies, where appropriate.

For *in-vitro* characterization of the NPs that were generated for transplantation, small aggregates of NPs were plated on poly-D-lysine (10 μg/ml) and fibronectin (5 μg/ml; both from Sigma) pre-coated cover slips in central well plates in NPM without growth factors. Half of the cultures were fixated in 4% PFA after 2 hours and stained for A2B5, nestin, Musashi and PSA-NCAM. The rest of the cultures were fixed after 7 days of differentiation, and stained for β tubulin III and GFAP. The cell surface markers NG2, O4 and GalC were stained in living cells followed by fixation in 4% PFA. The cells were incubated with primary antibody for 45 min followed by 30 min incubation with a secondary antibody. Mounting medium containing 4V, 6-diamidino-2-phenylindole (DAPI; Vector, Burlingame, CA) was used for nuclei counter staining.

For characterization of the *in-vivo* location and differentiation of the transplanted NPs, double immunofluorescent stainings were performed on 6–8 μm axial frozen brain sections. Human cells were identified either by the expression of human specific mitochondrial antigen, human nuclear antigen, direct GFP fluorescence visualization, or by an anti-GFP antibody. The sections were incubated with primary antibody overnight at 4°C followed by 50 min incubation with a secondary antibody at room temperature.

Images were taken by a fluorescent microscope (Nikon E600, Kanagawa, Japan) or confocal microscope (Zeiss, Feldbach, Switzerland). Three hundred cells were scored within random fields at ×1000 magnification. The percentage of each cell phenotype was determined by dividing the number of positively stained cells by the total number of GFP+, DAPI stained nuclei.

***Co-cultures of hESC-derived NPs and LNCs:*** Lymph nodes were excised from naïve mice. LNCs were cultured as single-cell suspensions, as described previously [10] with 2.5 μg/ml ConA or in control medium. NPs were irradiated with 3,000 Rad for 1 minute and then added directly to the LNC culture medium with non-stimulated or stimulated LNCs (10^5^ NPs/2×10^5^ LNCs)

***In-vitro proliferation assay:*** The proliferation of LNCs after 72-hour incubation *in- vitro* was evaluated by means of a standard ^3^H-thymidine incorporation assay, as described previously [10]. In all fluorescent activated cell sorter (FACS) experiments, cells were pre-coated with anti–mouse CD16/CD32 (BD Pharmingen,) to block unspecific binding, and T cells were identified by cell-surface labeling with APC-labeled anti-Thy1.2 (BD Pharmingen). All samples were analyzed in a FACSCalibur apparatus using the Cell Quest software (BD Biosciences, San Jose, CA). The proliferation of T cells obtained from naive mice was evaluated by FACS analysis for the incorporation of the cell division tracking dye 5(6)-carboxyfluorescein diacetate succinimidyl ester (CFSE), as described previously [31]. For CFSE FACS analysis, LNCs were pulsed with 3 μM CFSE (Molecular Probes, Eugene, OR) for 10 minutes, washed, and further cultured with or without ConA for 72 hours. CFSE-labeled, non-activated cells were used as control samples. The fraction of T cells that entered cell cycle was calculated by the formula:
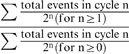
T-cell activation was analyzed by staining with PE-labeled anti-CD25 (Serotec, Bicestar,United Kingdom) for interleukin-2 receptor α (IL-2Rα).

***Statistical analysis:*** Clinical evaluations of EAE mice and quantification of the pathological features were performed by examiner, blinded to the experimental group. The results are presented as mean±SD. For comparison of clinical and pathological parameters between experimental and control groups, student's t-test was used. For analysis of the relationship between the inflammatory process and the tissue damage, regression analysis was used.
